# Into the Dark Domain: The UK Web Archive as a Source for the Contemporary History of Public Health

**DOI:** 10.1093/shm/hkv028

**Published:** 2015-06-03

**Authors:** Martin Gorsky

**Keywords:** methodology, websites, local government, public health

## Abstract

With the migration of the written record from paper to digital format, archivists and historians must urgently consider how web content should be conserved, retrieved and analysed. The British Library has recently acquired a large number of UK domain websites, captured 1996–2010, which is colloquially termed the Dark Domain Archive while technical issues surrounding user access are resolved. This article reports the results of an invited pilot project that explores methodological issues surrounding use of this archive. It asks how the relationship between UK public health and local government was represented on the web, drawing on the ‘declinist’ historiography to frame its questions. It points up some difficulties in developing an aggregate picture of web content due to duplication of sites. It also highlights their potential for thematic and discourse analysis, using both text and image, illustrated through an argument about the contradictory rationale for public health policy under New Labour.

## Introduction: the Dark Domain Archive

Public health researchers have long been interested in the internet as a medium of communication, focusing *inter alia* on web tools for data gathering, on dissemination of health messages and on the networking potential which the World Wide Web provides.^1^ Facilities for the capture of websites, for example, are now routinely included in qualitative data analysis software such as NVivo. So far this activity has largely passed historians by, notwithstanding their harnessing of computing power in database work and in the use of the Web to host digitised primary material.^2^ Readers of this journal with British history interests will be aware, for example, of sites such as Old Bailey Online, the National Archives' Hospitals Database, and the Wellcome Trust's ‘London's Pulse’ collection of Medical Officer of Health reports.

However, as the timespan since the inception of the Web lengthens, and the number of sites, both live and inactive, proliferates exponentially, new methodological questions arise about treating web content itself as primary documentation.^3^ Information technology scholars brandish extraordinary estimates: that before the internet the total data preserved throughout human history was the equivalent of perhaps 12 exabytes (one quintillion bytes), but that as mass computing spread this rose to some 180 exabytes by 2006, and exceeded the zettabyte mark (1,000 exabytes) by 2011.^4^ Alongside this hyper-abundance is the fact that global ‘technological memory’—to us, the written record—is now almost entirely digital: 94 per cent in 2007.^5^ What does this mean for scholars seeking to use websites as sources in documentary research? Sheer scale means they can hardly be expected to make a bespoke selection from the live web in their data collection. However, if they are to draw instead on new digital repositories, how should the material therein be stored, accessed, selected and approached?

Historically, the duty of documentary collection for the public realm has been intertwined with the growth of the national and local state, and the need to preserve official memory.^6^ Before the 1990s, documentary researchers trusted to these public agencies, alongside other institutional repositories, to collect and conserve single paper copies, selected according to some criteria of importance.^7^ The scale, ephemerality and democracy of the web pose challenges of a different order. The dominant player thus far has been the Californian non-profit organisation, the Internet Archive, which has been preserving web content since 1996. Its Wayback Machine periodically ‘crawls’ the web and captures for posterity any sites whose programmers have not incorporated ‘robot exclusion’ text to prevent such automated collection.^8^ Meanwhile, the major national repositories have been considering how to fulfil their obligations as collectors of the official record, and of other material considered to be of future significance. Not only do they face the practical problems of acquiring data, but in making these available, officials must also negotiate the evolving parameters of copyright and legal deposit legislation for digital material.

One such repository is the British Library (BL), which is increasingly making available to researchers its UK Web Archive of .uk domain sites.^9^ These are sites whose domain names were registered in the United Kingdom, and which the BL thus deems within its collecting remit. Their URLs have suffixes such as: co.uk (businesses, firms); ac.uk (academic institutions); gov.uk (national and local government); org.uk (civil society organisations); and many others, for example nhs.uk (those of the UK National Health Service (NHS)). In creating this dataset the BL has taken a twin-track approach, beginning with the selective capture of individual sites for which permissions are negotiated with interested parties. Because of the cumbersome processes of justification, selection and consultation involved in this, the BL, supported by the charity Jisc, has also procured from the Internet Archive all UK domain sites captured between 1996 and 2010.^10^

Formally entitled the JISC UK Web Domain Archive, this extends to some 35 terabytes of data, or 2.5 billion items, and far exceeds in scale those collected by selective capture.^11^ Copyright restrictions and the challenge of managing access meant that it could not be put immediately into the public domain, hence the adoption of the colloquial term ‘Dark Domain Archive’ (DDA).^12^ The BL began work in 2011 to create a search engine through which researchers would ultimately access and use this resource. A prototype provided access to a 12 per cent sample of the DDA, enabling users to search by subject and providing both access to the archived sites and some aggregated results of different types of search. In 2012, in collaboration with the Institute of Historical Research, the BL's ‘Analytical Access to the Dark Domain Archive’ initiative invited interested scholars to bring research ideas for small-scale trials of this interface.

The present article reports the results of one such trial, undertaken in 2013–14, whose subject is web representations of public health in English local government. There are five sections: an outline of the historiographical context; a description of research questions and methods; a summary of methodological challenges and opportunities associated with selection from the archive and with analysis of a corpus of websites; some illustrative findings; and finally a discussion of implications. It should be stressed that the main aim is to introduce readers to methodological aspects of using the UK Web Archive search engine. For reasons that will become clear, the substantive results generated must be considered provisional. Nonetheless, they are presented here to encourage discussion of the website as a distinctive type of documentary source, with which contemporary historians now need to engage.

## Public Health in Local Government: Past and Present

The research questions for this DDA trial project were triggered by recent changes to the place of public health in Britain, following the Health and Social Care Act of 2012. This legislation is best known for the changes it wrought to the structure of Britain's NHS, bringing to fruition the policy trajectory begun in 1989 of introducing an ‘internal market’ to the service.^13^ The 2012 Act abolished the last tiers of state planning structures to leave a putatively autonomous and self-correcting system.^14^ One outcome of this was the relocation of public health, which was moved from the now defunct NHS bodies into local government.^15^ This provoked scepticism that it was not positive policy, but essentially a default option. It may also have resulted from horse-trading within a fractious Coalition government, whose minority Liberals, struggling with backbench dissent, harboured a localism agenda. As Timmins recounts, ‘… the Conservatives “had to have something to give the Lib Dems”’ in order to secure the legislation.^16^

The relocation was, however, not exactly new. Historically, British public health had been a duty of the councils of municipal boroughs, counties and districts since its emergence in the mid-nineteenth century.^17^ Local health departments led by a prominent executive, the Medical Officer of Health (MOH), remained in place until 1974, when the function was brought into the NHS as part of a major health service reorganisation. Prior to the 2012 Act though, policy rhetoric emphasized a new momentum towards localism. Politicians and officials argued that by situating public health in local authorities there was a chance to address social determinants of health through working alongside other local government departments; close knowledge of local circumstances would hasten the reduction of health inequalities; the ability to affect built environments—‘shaping local places’—would also foster healthy living; and on top of all this, grassroots democratic input would be enhanced.^18^ Yet it was striking to note that policy makers in 2012 made no reference to this earlier history and its implications for the new agenda, other than the Department of Health's fleeting allusion to ‘… returning public health home …’ and ‘… reviving “a long and proud history …”’.^19^

The contradiction between this rhetoric and public health's contested historiography is striking. Few would deny that the Victorian era of sanitary reform merits the ‘long and proud history’ accolade, even if scholars still argue about its contribution to rising life expectation.^20^ The claim that the early twentieth century was a ‘golden age’ when local government health departments exercised an effective range of services is more hotly disputed.^21^ Municipal medicine may have been at its zenith, but the charge sheet of MOH failings is also long, notably of administrative overstretch to the neglect of prevention, and of ineffectiveness as watchdogs of the poor during the depression.^22^ As for the advent of the NHS, the isolation of public health within the new tripartite structure had drastically curtailed the role of the local state and ushered in a period of ‘decline’.^23^ However, a strong case has also been made that the MOHs were authors of their own downfall.^24^

This critical literature on the 1948–74 period makes two interrelated points relevant to recent policy: first that the rationale for locating public health in local government became increasingly untenable, and second that the MOHs failed to articulate a new role in changing times.^25^ Declining infectious disease mortality redirected attention to ‘chronic’ illnesses, yet it was the central state and the voluntary sector that increasingly made the running in advocacy and ‘health education’.^26^ Sanitation slipped from the agenda, and after the Clean Air Act (1956) environmental concerns became separated from medicine. Social work departments encroached on established remits like community care for older people and psychiatric patients.^27^ In the face of this, it is argued, the local MOH proved slow to adapt, failing to develop and proclaim a new philosophy of public health. This lack of vision was the more regrettable given the vigour of academic ‘social medicine’, based on chronic disease epidemiology and social determinants of health, which could have provided inspiration.^28^ But the traditionalist and service-oriented MOHs failed to provide leadership, so that by 1974 they seemed to have exhausted their usefulness, and the local government setting appeared irrelevant.

This pessimistic view of the past underscores sceptical understandings of recent policy. However, is the reading too bleak? Some elements of the literature offer a more positive interpretation. On the one hand, this stresses the degree of choice available to citizens, viewing variable patterns of expenditure as desirable expressions of localism.^29^ On the other hand, it draws on urban case studies to trace the creative efforts of local MOHs in developing new initiatives.^30^ In the more proximate contemporary history, the narrative is one of revival. This was assisted by the international currency of ‘New Public Health’, centred on promoting healthy lifestyles and addressing the ‘upstream’ environment, in which these could flourish.^31^ Closer to home, food poisoning scandals and the appearance of HIV AIDS prompted the revival of public health infrastructure, with the recreation of the MOH role as the Director of Public Health (DPH), now located within regional and local NHS bodies.^32^ Big-government stewardship returned in the enactment of tough anti-tobacco legislation. But there was also a revival of public health in its localist setting, with official encouragement of closer working with local government, first in an ad hoc way, then in 2006 with legislation that permitted joint appointments of DPHs across NHS and local government.^33^

## Research Questions and Methods

In sum then, does this recent history imply a coherent policy trajectory, representing continuity with ongoing trends and a new vision of a localist public health? The DDA can help address this at least in part, by revealing how policy was represented in the years immediately preceding the reform. The prior history of public health in local government suggests that the DPHs should be at the heart of the investigation, for the MOH/DPH role has always been the pivot on which success or failure turned. So it is above all their web presence that is of interest.

Thus the three questions driving the study are: first, what was the chronology and geography of web representations of public health in local government? These are essentially empirical problems for which the aggregating functions of the search interface seemed well suited. Second, how were benefits of localism and joint working between NHS and local government represented by DPHs? If a coherent, grounded policy was discernible, at least in presentation, then the case for viewing the 2012 Act as positive continuity would be strengthened. Third, did DPHs use the Web to provide a clear rationale for public health in local government? This question arises directly from the criticism that pre-1974 MOHs failed to develop and articulate a compelling vision. How did the later DPHs meet the same challenge?

Given the small-scale nature of the project, the chosen method was to select websites that contained the term ‘director of public health’, and which demonstrated the convergence of public health and local government through electronic links: either ‘nhs.uk’ sites with links to ‘gov.uk’ sites, or ‘gov.uk’ sites with links to ‘nhs.uk’ sites. Because the trial version of the DDA contained only some 12 per cent of the actual archive, the number of sites retrieved using this sampling frame was apparently 520, according to the summary counts by year. The numbers generated between 1996 and 1999 were so small that the analysis concentrated only on sites from 2000 to 2009.

Within the limited timeframe for the project, it seemed reasonable to carry out both quantitative and qualitative content analysis. A database was created recording the following variables: the site's URL; owner; number of duplications; links; keywords; purpose; and key themes. In the process of recording these sites, any qualitative data deemed relevant to the research questions was selected and captured. This material was then subjected to three analytical processes. First, a thematic analysis to establish the basic empirical detail required to address the questions. Second, a discourse analysis, to identify recurrent tropes germane to the questions of public health vision, and to consider the genealogy of such discourses. Third, analysis of the sites as visual artefacts, for scoping indicated that many contained digital artwork and imagery, which addressed spectators in certain ways and expressed certain meanings alongside or against written text.

**Fig. 1:**
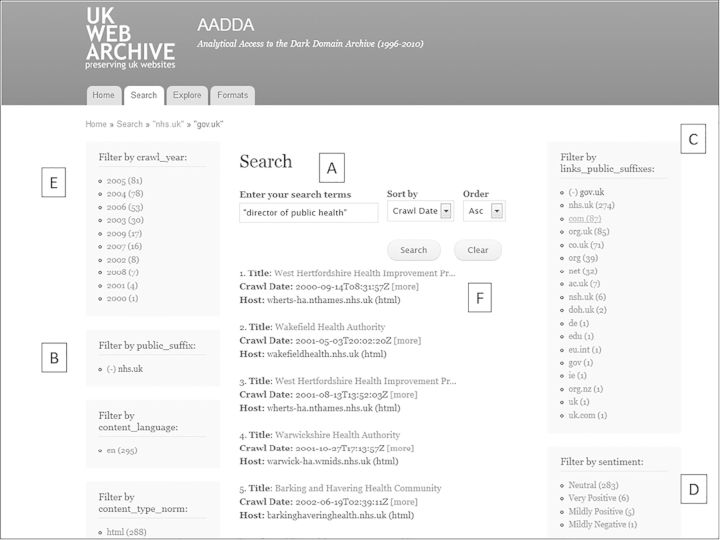
Dark Domain Archive search interface, April 2014. Reproduced by permission of British Library.

An example of the search interface is shown in Figure 1, annotated with letters A–F to indicate key features. The search term, ‘director of public health’ is entered (A) and the results (F) generated below. (Although not used here, the search facility also allows for combinations of terms, exclusion of terms, proximity searching, where the maximum words separating the two terms can be set, and so on.) The results can then be refined by suffix (B), and here the filter has limited the results to ‘nhs.uk’ sites, in other words websites produced by NHS organisations such as primary care trusts or health authorities. Next the selection can be further refined by filtering by links (C), so that for example if the ‘gov.uk’ option was chosen, the search would return only those ‘nhs.uk’ websites containing electronic links to, say, Department of Health or local government websites. Another option is to filter by ‘sentiment’ (D), drawing on a technique of big data analysis pioneered by commercial or political users; this aims to gauge public opinion from a large number of websites by counting words which indicate either positive, negative or neutral sentiment (for example towards a particular product or slogan).^34^ There are various other filters, some absent from this screenshot because they lie lower down the webpage, and these include postcode (where an address is given on the site) and language. Finally, and most crucially for the historian, a filter by year (E) can then be applied.

With each application of a filter the resulting number of websites captured is shown alongside the various categories, and these aggregate counts can, in principle, be used for preliminary analysis such as time trends or spatial distribution. Then, once the chosen filters have all been applied and the resulting selection is listed (F), the researcher simply clicks on the link shown at ‘Title’ and the archived site appears.

In the present project some peculiarities became apparent at the scoping stage when the initial time trends were produced. It seemed reasonable to assume that the time series data would bear some relation to the overall growth of the UK domain (the total number of websites ending in the .uk suffix). According to ‘nominet’, the organisation that records the new registrations of site names, the pattern was one of unbroken expansion. Overall the registry grew progressively from about 1 million names to some 11 million by 2012. Growth was most rapid 2001–07, the rate peaking at about 60 per cent per annum in 2005, before slowing 2008–12, falling close to 10 per cent by 2009.^35^ It seemed probable that the number of public health themed sites would broadly mirror this continuous upward trend, although perhaps with some sensitivity to the timing of key issues in popular and political discourse, such as the smoking ban (2007), the swine flu scare (2009), or the introduction of the new DPH joint appointments policy (from 2006).

**Fig. 2:**
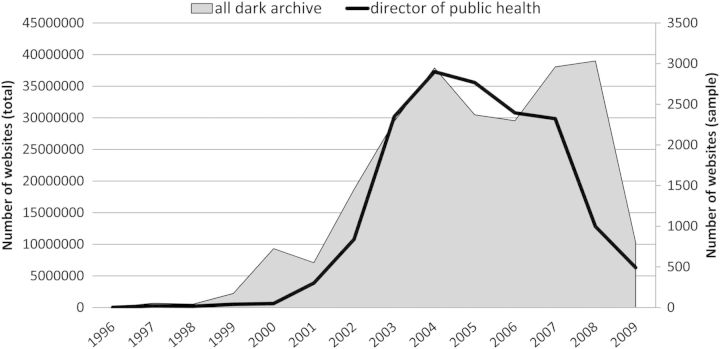
Total Dark Archive, and sample of sites containing ‘director of public health’ (2nd y-axis), 1996–2009

**Fig. 3:**
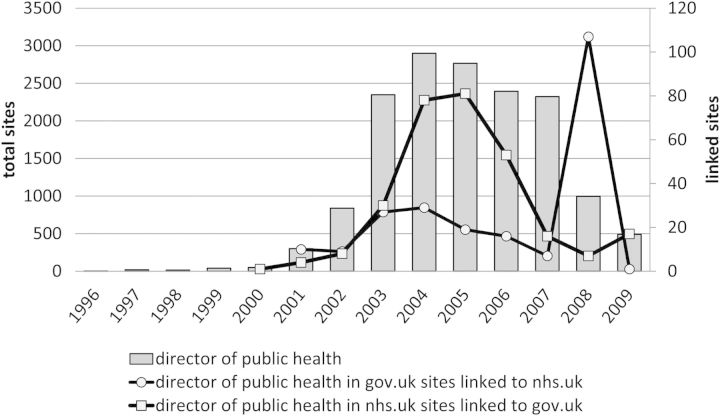
UK websites containing “director of public health”, total and linked gov.uk/nhs.uk, 1996–2009

Surprisingly, when the samples were generated and the time trends graphed, the pattern was nothing like this. Figure 2 shows all .uk domain sites (i.e. not just nhs.uk or gov.uk sites) containing ‘director of public health’, and the pattern was one of rapid rise to 2004 then slow descent, which seemed counter intuitive. This prompted examination of the size of the whole Dark Archive (the area block) with its millions of sites, and in fact this time trend was rather similar to that of the sample. Next, by applying the filters, the time trends for the subsidiary samples of ‘nhs.uk’ and ‘gov.uk’ sites were derived (Figure 3), and again these followed the pattern of rise and slow descent, apart from an unexpected peak in 2008 of ‘gov.uk’ sites. These initial results raised the possibility that the time trends generated by the search engine were an artefact of the collection process used by the Internet Archive's Wayback Machine. This in turn may have biased the sample in some way.

## Methods: Challenges and Opportunities

The preliminary concern was therefore to resolve this issue. A logical starting point was to investigate the anomalous peak of linked gov.uk sites in 2008 (Figure 3), which the search interface showed to consist of 107 sites. Scrutinising the sites themselves, of which there were in fact only 77, revealed that they came from only two local authorities, Wigan and Wolverhampton. This selection contained a mere eight unique pages, alongside 13 duplicates and 56 whose link led to the Wayback Machine's ‘not found’ page. This was the first evidence of selection bias caused by duplication, and of bugs in the DDA.

**Fig. 4:**
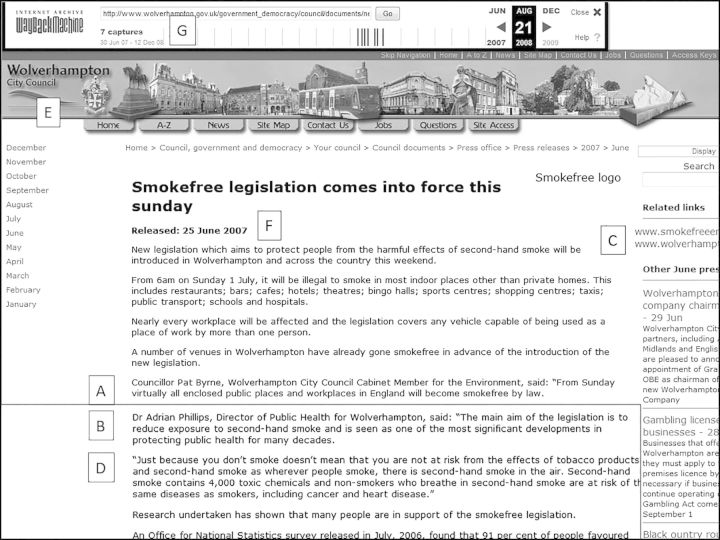
Press release Wolverhampton City Council, 2007

Source: <http://web.archive.org/web/20080112154036/http://www.wolverhampton.gov.uk/government_democracy/council/documents/news/press_releases/2007/june/250607a.htm>, accessed April 2014. Reproduced by permission of Wolverhampton City Council.

That said, of the remaining eight pages all spoke usefully to the research questions: one spelt out the public health duties of a council Director for Environmental Services; others showed the Wolverhampton DPH in joint working with the Chief of Police to address kerb crawling, and with the Chief Executive for Environmental Services on the smoking ban; others showed the Wigan DPH leading a multi-agency forum on suicides, and regularly attending Council Overview and Scrutiny Committee to give a presentation on PCT efforts to improve ‘health and wellbeing of the people of Wigan’; these meetings also included a regular report on health inequalities in the city. One such site is shown in Figure 4, again annotated with letters A–F to indicate salient points; the lower section of text has been cropped to include key passages within this screenshot. Produced by Wolverhampton City Council, it is a press release on the implementation of the smoking ban.

How might such a document be approached? Surface thematic analysis yields empirical evidence of the smoking ban as a spur to joint working between NHS and local government. The principal actors are the council member with environmental control responsibilities (A) who announces the legal framework, and the DPH (B), who provides the health justifications. The web links (partially obscured in the screenshot) to the NHS primary care trust (PCT) (C) make manifest electronically the integration of different public bodies in this action. The text contains various elements (D) for consideration in discourse analysis, such as its discussion of risk and protection, its deployment of epidemiological terms, and its appeal to public opinion. These invite reflection on the genealogy of such language and the work it does in sustaining the exercise of judicial power. As a visual artefact, the website adopts a conventional format with its standardised header of juxtaposed images (E) under which the text nestles. These address the viewer as citizen, mobilizing themes of civic power, urban pride, thrusting modernity and manufacturing heritage. Finally, the date of the press release (F) is 2007, despite its appearing in the 2008 DDA search results. On the Wayback Machine banner at the top, a total of seven captures are recorded (G), indicating that between June 2007 and December 2009 there were multiple harvests of this site.

Working further into the sample, the scale of this problem of duplication became apparent. In addition to those recorded on the Wayback banner, there were other effectively identical pages, but with minute differences, that were thus not automatically recorded.^36^ Moreover, the crawl dates were not, as might be expected, at even intervals. Comparison of duplicate sites revealed, for example, that some duplicates seem to have been captured on consecutive days.^37^ Clicking through on a ‘captures’ link on the Wayback banner (Figure 4, G) takes the reader to a page showing the underlying crawl timings. This reveals, for example, one frequently captured site, from West Lincolnshire PCT, was archived 137 times between 2002 and 2014.^38^ Moreover, an examination of the calendar of crawls revealed a highly unpredictable sequence, with 10 captures occurring in a single month, January 2006.

What all this means is that the number of unique sites—the documents of interest to the researcher—is significantly lower than the total number apparently returned through the search. For example, taking the ‘nhs.uk’ sites with links to ‘gov.uk’ for the period 2001–03, only a small number, 34, were returned by the selection process, and of these only 17 proved on inspection to be unique. For 2005, the peak year, there were 51 unique sites out of 81 returned (the Wayback capture data showed that the 30 duplicates appeared 330 times in the archive across all years). Only in 2009 was the problem minimal, with a single duplicate from 17 sites. As Figure 2 indicated, the DDA holds considerably fewer sites in that year, and this is related not to the actual volume of .uk domain sites on the internet in that year, but to the more aggressive ‘de-duplication’ applied by the archive managers.^39^

With respect to the postcode function, and the potential offered for spatial analysis, the promise that the aggregating functions in the search engine would yield robust preliminary results was not borne out. Leaving aside the difficulties of duplicates, it was noticeable on inspection of the sample that geographically the results came from a comparatively small number of local authorities and health trusts/authorities. Whether this is because these were indeed the few pioneers, or is simply an artefact, either of the crawl process, or of the composition of the initial 12 per cent sample of the DDA, is impossible to say. With respect to this project though, it did raise questions about representativeness and hence the validity of any conclusions drawn.

As for sentiment analysis, this tool in the search engine did not appear particularly useful. Possibly this was because the method does not lend itself well to the formal administrative language found in the sites considered for this question, which were all pages generated by official bodies. ‘Neutral’ was by far the dominant sentiment recorded and where it was not there was little to be gleaned. In 2005 for example, in the ‘nhs.uk’ sites, the search engine counted only four ‘Very positive’ sites, of which three were duplicates. Once the unique page was scrutinised it proved to be a self-valorising press release, containing words like ‘excellent’, ‘praised’ and ‘successes’.^40^ This was interesting in itself, although the site was seeking to cultivate sentiment, rather than reflect it.

Finally, it became apparent that following a strict sampling strategy might exclude vital documents. A particularly relevant source located in the search was a booklet precisely on the question of joint working, in the period prior to the formalisation in 2006 of DPH appointments in local government.^41^ However, this was only traced by following a link from a Public Health Network NHS News page. The Wayback Machine, it should be stressed, seeks to archive a complete site, including its links, but in practice only some of these remain live. So paradoxically, while historians will often have to apply a tight sampling frame to rationalise the vast number of websites that potentially speak to their questions, they will also need the flexibility to depart from this if links throw up vital ‘gems’. For this type of question an incremental process that gradually develops the search terms and sampling frame will be most appropriate.

In summary, while some of the methodological difficulties described here arise from working with a prototype search engine and a small sample of the DDA, others attest to larger issues in website archiving and selection. It is extremely helpful to researchers to have an aggregating function in the search interface so that they can develop time series and spatial analysis as a prelude to plunging into the web content itself. Yet the volume of duplicate sites and bugs rendered these summary numbers meaningless in the present project. Even assuming all the ‘not found’ and broken pages can be removed, the problem of duplicate handling remains. On what basis should de-duplication proceed? Should all duplicates be removed leaving only the website at the time of its first harvest from the web? Should all duplicates within a given year be removed, allowing at least a cross-sectional analysis on an annual basis? Ideally researchers should have a range of options to suit their question.^42^ Before decisions are made more needs to be known about the crawl process itself, as this was apparently not a neat, systematic activity occurring at regular intervals.

In this project then, systematic quantitative analysis was not possible using the initial calculations performed by the search interface. It can be done, but only with manual weeding of duplicates, requiring careful inspection and time-consuming comparison. In the event the 12 per cent sample in the DDA proved too small for establishing chronology and geography with confidence. Nonetheless, in working through the unique pages that were returned, and following a flexible strategy allowing for collection beyond the sampling frame, much apposite material was collected.

## Websites as Representations of Public Health in Local Government

This section presents the findings, although given the caution about representativeness, they are offered as much for their methodological contribution as for their substantive importance. In light of the content analysis adopted here, what do they suggest about the distinctive features of websites as historical documents?

Coming first to the thematic analysis, the aim was to identify empirical material to show what forms of localist public health were evident and how the benefits of ‘joined-up’ working were represented. One clear theme cutting across various sites was that some joint working between NHS and local government had what might be called a ‘service logic’, impelled by a need to work across the health/social care boundary. In other words, there was a need to integrate services for which the two sides had separate responsibilities and budgets, but across which individual patients required continuing care. Specifically this referred to older people with complex needs, psychiatric patients and people with learning disabilities. However, this is nothing new, for the challenges of working across the health/social care boundary have preoccupied the British welfare state at least since the ‘bed-blocking’ controversies in 1950s geriatric medicine. An established literature has identified issues such as the status and knowledge asymmetries between NHS and local government employees, and the financial incentives to cost-shunt and work independently rather than to collaborate.^43^ Thus a ‘service logic’ is as old as the NHS and cannot be considered a decisive factor here.

Instead, many sites suggested that more specific to the time period were collaborations founded on what might be called a ‘New Public Health logic’: that is where the need to work with local government arose from a desire to implement policies designed to affect ‘lifestyle’ diseases. Recurrent issues evident throughout different sites were: smoking cessation, where the ban raised environmental and public order issues, and work in schools furthered health promotion; diet and exercise, where again the linkages fostered work with schools, and the shaping of the built environment around salutogenic goals; active lifestyles, where the links aimed to infuse transport and housing with health considerations, or larger environmental schemes, for example to develop sports centres; and alcohol and drugs, where, as with smoking, there were public order issues, and also the role of social services in drugs treatment and aftercare, work with schools to educate and inform and so on.

What became questionable though, as more and more sites were considered, was the extent to which these were grassroot initiatives inspired by the local DPH. Instead, the presentation of partnership policies to further public health seemed to emanate more or less directly from central government efforts to regenerate local action. This was sometimes made overt through references on different pages to source ‘New Labour’ policy documents, which were then examined to verify the linkage.^44^ Particularly influential were the ‘NHS Plan’ of 2000, the White Papers of 1997 and 2004 and the Local Government Act of 2000.^45^

This observation is underscored by the results of the discourse analysis, which builds on the attention paid to language in constructing the dataset, observing recurring jargon or keywords that figured across sites. Indeed, a striking feature of reading websites as historical sources is their amenability to this approach. Although generated by diverse individuals in different locations, it is possible to observe common languages circulating through the sites, whose very succinctness makes them easy to identify. Two of these might be described as ‘partnership’ discourses, concerned to constitute social and political relationships within particular organisational structures, and thus speaking to the second research question, of how DPHs represented the value of joint-working. Another two can be described as ‘public health’ discourses, which legitimise the medicalisation of the social realm in these local practices, and so speak to the third research question, about rationale and vision.

The first ‘partnership’ discourse is loosely administrative, incorporating tropes like: ‘co-operative’, ‘partnership’, ‘seamless service’, ‘real collaboration’, ‘working closely’, ‘shared’, ‘integrated’, ‘together’, ‘partnerships … with local stakeholders’, ‘NHS family’, ‘coordinate care’; qualifying terms sometimes included: ‘modernisation’, ‘planning’, ‘healthy strategy’. The second ‘partnership’ discourse is found in terms that signal varieties of public engagement: ‘take part’, ‘local community’, ‘involving people’, ‘views count’, ‘help shape the future’, ‘entitled’, ‘plans’, ‘knowledge’. Here then, the partnership is between institutions and public. The two ‘public health’ discourses meanwhile are rather contradictory. One is oriented towards the personal, situating health within a framework of ‘risk’ and ‘choice’, and interpellating the reader with individualised addresses: ‘better health’, ‘prevent’, ‘advice’, ‘help’, ‘aware’. The other ‘public health’ discourse alludes to inequalities and injustice, positioning health as a collective outcome of economic forces. Here signal terms seem implanted from social epidemiology: ‘reducing health inequalities’, ‘social exclusion’, ‘inequalities’, ‘disadvantage’, ‘regeneration’, ‘deprivation’, ‘inequalities of access’, ‘desperate health problems’, ‘joint working to improve health inequalities’.

As suggested, these may be treated as languages emanating from governing elites that were adapted by local officials, and in part they reflect the downward cascade of innovations from Whitehall. Their likely source is the various policy documents of the late 1990s and early 2000s generated by the Department of Health and senior Labour politicians, in which ‘partnership’ was articulated as a distinctive welfare policy.^46^ In health, the problem was to marshal a political language through which to prosecute a policy that essentially continued the Thatcherite trajectory (of market disciplines, provider pluralism and new public management) while reviving the more solidaristic elements (and later, heightened funding) which voters expected of the centre left. An early step was to propose a ‘… ‘third way’ of running the NHS—a system based on partnership’.^47^ New Labour had nominally accepted the critique of the NHS as a ‘command and control’ relic of big government hatched under postwar austerity and now incompatible with consumer capitalist society.^48^ Yet it also rejected the internal market, at least at the rhetorical level. Replacing ‘competition’ and ‘fragmentation’ with a language of ‘co-operation’, ‘partnership’ and ‘coordination’ softened the dynamic, although in essence commissioning was retained.^49^ At the same time it legitimized planning, which was reintroduced in the (generally effective) form of ‘targets’ regimes, although now minimising big government connotations.^50^

Labour had a similarly problematic relationship to health inequalities as it sought a new public health agenda. Blair, Milburn, and others such as the influential adviser and bank chief Derek Wanless, accepted the consumerist premise which emphasized public health as individualized ‘healthy choices’ rather than state stewardship.^51^ Such a position was also compatible with tenets of moral reformation infusing Third Way politics, which drew from communitarian thought the primacy of individual responsibility.^52^ They were, for example, pledged to work with business rather than restrict it in areas like alcohol pricing or junk food curbs.^53^ However, progressive elements in both the party and the public health community (represented here for example by the Wolverhampton and South Gloucestershire DPHs) were anxious to stress a health inequalities agenda.^54^ This had emerged in the 1970s, driven by politicians like Barbara Castle, and academics like Peter Townsend and Jerry Morris, but then lain fallow under Thatcherism (with the purported suppression of the Black Report in 1980 the portent of Conservative neglect).^55^ Thus an uneasy contradiction arose for a centre left party, played out in the competing discourses observable in the local sources.

In sum, the cross-cutting discourses surfacing in the websites seem to derive from these political languages, which were formulated to address a particular moment in the political economy of Britain. A Labour government had been elected to restrain the neo-liberal trajectory launched by the Conservatives, but not to reject its central tenets. Its health policies, and the texts through which these were presented, negotiated this contradiction.

**Fig. 5:**
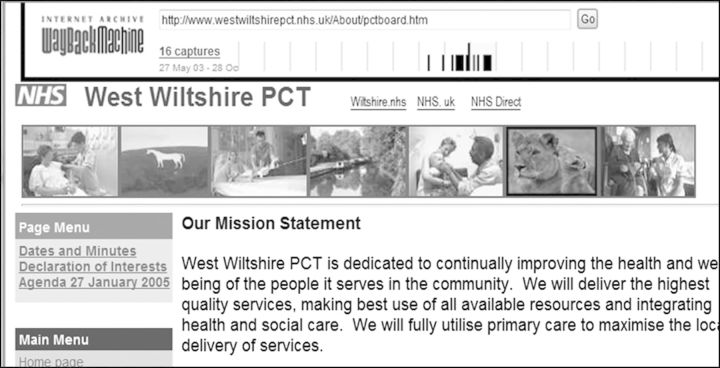
West Wiltshire PCT, ‘Our Mission Statement’, 2005, <http://web.archive.org/web/20050205132833/http://www.westwiltshirepct.nhs.uk:80/About/pctboard.htm>, accessed January 2015. Reproduced by permission of NHS Wiltshire CCG (www.wiltshireccg.nhs.uk).

Turning finally to the analysis of websites as visual artefacts, there are some interesting discourses circulating here too. One recurring trope in the selection of illustrations is the juxtaposition in header banners of tradition and modernity, the former expressed through local scenes of buildings, monuments or industries, and the latter with new health infrastructure. In this imagery, it is important to note, ‘health’ is synonymous with medicine. The dominant visions are of curative interchanges between patient and uniformed professional, in other words of the sick, not the healthy, individual. Figure 5 is a characteristic example, from an NHS trust with a strong reputation for joint working.^56^ Here it has branded itself as a community asset by interspersing such depictions (in this case aligning ‘primary care’ with ‘services’) with those that evoke local history, or more accurately, heritage. The signifiers are: the Westbury White Horse, a hill figure cut in the chalk downs of Saxon Wessex; a barge set on one of Wiltshire's canals, here connoting bucolic leisure rather than urban commerce; and the ‘lions of Longleat’, a stately home whose grounds were adapted into a safari park in the 1960s.

Rather than intentionality, the prevalence of such motifs may primarily reflect the limits to design skills in the early years of the Web, and the nature of images to hand for the first cohort of digital designers. Nonetheless, if these constructions are approached from the perspective of reception, the key question is how such assemblages work upon the viewer. The association of the NHS with comfortable images of stability and continuity, at once evoking a wider Britishness and a proximate rural neighbourhood, calls upon the spectator to regard health services as common culture. This encoding also aligns closely with the political language of New Labour, for such juxtapositions were central to the ‘Third Way’ analysis of the NHS. This argued that the service was one of the greatest creations of Old Labour, but was now in desperate need of ‘modernisation’—temporarily a prime buzzword of British politics.^57^ At the same time, through configuring ‘primary care’ as an individualised encounter with a white-coated expert, the viewer's sympathies are solicited in the interest of cure, rather than prevention; it is medicine's claim to ‘available resources’ that is underscored, not public health's.

**Fig. 6:**
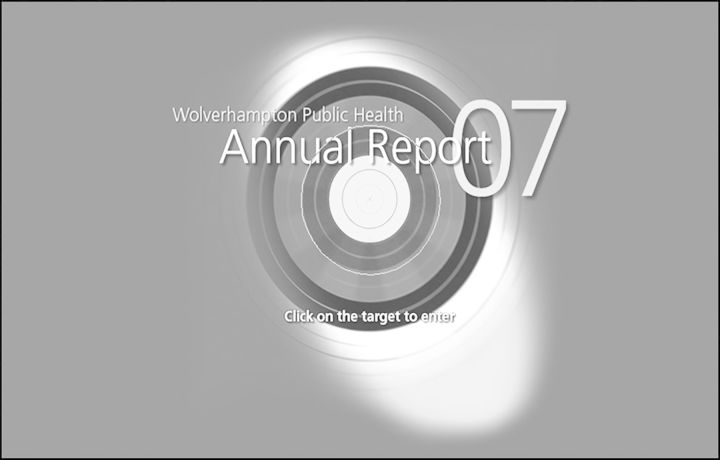
Wolverhampton Public Health Department, ‘Annual Report 07’, <http://web.archive.org/web/20090618043627/http://www.wolvespct.nhs.uk:80/phar/index.html>, accessed April 2014. Reproduced by permission of Wolverhampton City Council.

As for visual material specific to public health discourses, a burgeoning potential for artistic communication became more evident in sites drawn from the final years of the sample. This was when graphic design began to adopt the interactive approaches offered by the medium. Figure 6 is the homepage of a DPH annual report, again from Wolverhampton, which emerges from the sources as a particularly vigorous council and health department. Quite unlike the traditional banner images already discussed, it utilises the interactive functionality of the web as an aid to social marketing. Here the ‘target’ motif that provides the entry portal to the report's pages does a double duty. It hails the viewer primarily as individual subject, rather than as local citizen, and it succinctly captures the goal orientation required of the health conscious reader, alert to the risk factors that the DPH strives to communicate.

**Fig. 7:**
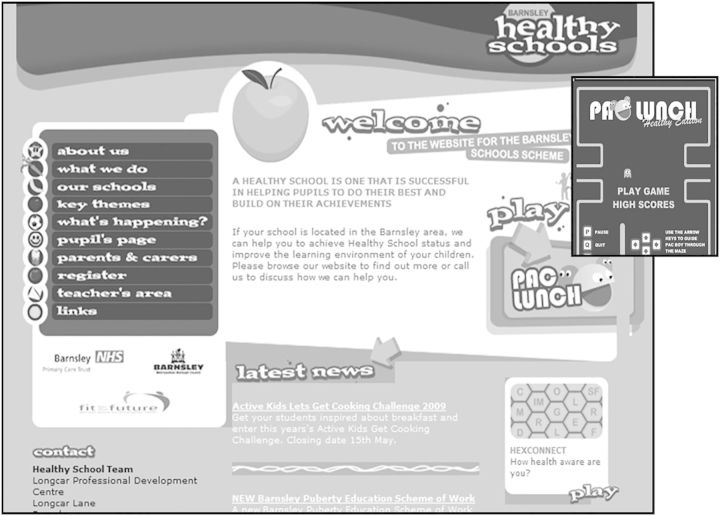
Barnsley NHS PCT/Barnsley Borough Council ‘Barnsley Healthy Schools’, <http://web.archive.org/web/20090503215041/http://www.barnsleyhealthyschools.org.uk:80>, accessed April 2014. Reproduced by permission of Barnsley Council.

A final example, Figure 7, again testifies to the creative dynamism infusing web design in another authority with a reputation for excellence in joint working.^58^ Now colour, images and fonts are resonant of websites such as virtual worlds, or gaming software used in other platforms. Employing child-oriented design, the Barnsley Healthy Schools campaign packages its message about nutrition in a format legible to its target recipients, who are addressed as participants in the school system, whether as parent, pupil or teacher. It even employs interactive games (‘Pac-Lunch’, inset), through which children can absorb health messages. Such sites raise a host of questions. Health researchers will wonder about the effectiveness of these interventions, and psychologists about their implications for the mind. For public health historians this is the next chapter in a process that dates at least from the 1970s, of the application of social marketing techniques to new media to promote health.^59^ It also marks a further step away from public health conceived as ‘the organized efforts of society’, to one in which (to quote Conservative leader David Cameron) it is ‘ultimately of course … about individual behaviour. … it's up to individuals and families to make their own choices.’^60^

## Discussion

The aim of this study was to consider methodological aspects of website analysis through a contemporary historical case study. It drew on the Dark Domain Archive as a repository, and posed three questions about the recent development of public health in Britain. The starting premise of these was that historical amnesia had attended the recent return of the public health function to local government. Consideration of historiographical debates about its performance there in earlier periods suggested that the rhetoric surrounding the latest policy merited interrogation. Critical scrutiny of web representations could contribute to this, and also provide a test case for exploring web pages as primary sources. In anticipation of a potentially large amount of data that might overwhelm a small, time-limited project, a narrow search strategy was pursued, although with some flexibility to depart from this if links promised highly relevant documents.

On the first question posed, about the chronology and geography of web representations of public health in local government, no clear answer was obtained. This was partly related to developmental aspects of the archive, such as a geographical selection bias in the sample, and the presence of errors. It also suggested an intrinsic challenge, that of duplicate sites, for past crawl processes appear to have been irregular and thus to have captured numerous identical, or near-identical, pages. This meant that the aggregate figures produced by the search engine were misleading. Nonetheless, the evidence from numerous unique sites made it quite clear that the rapprochement between public health and local government was more than the forced outcome of legislation in 2012. It predated this, and had its own determinants.

On the second question, of how the benefits of localism and joint working were represented by DPHs, the sources came into their own. Web pages are relatively succinct, and the reader soon observes significant commonalities between texts and images irrespective of authorship or place. Content analysis suggested that two logics were impelling joint working at a local level, the ever-present problem of integration across the health/social care boundary, and the more pressing ‘new public health’ challenge of addressing cardio-vascular diseases and cancers considered to be lifestyle-related. Some of the discourses through which these were presented were optimistic and aspirational, positioning ‘partnership’ and localism as ‘modern’, empowering forces. Close attention to their sources identified them with the political language developed by New Labour to adapt centre-left mobilisation to a post-Thatcher polity.

The framing of local initiative within this larger rhetoric was also germane to the third question, of whether DPHs were using the web to provide a coherent rationale for public health in local government. Here the consideration of images alongside text pointed to areas of inconsistency. Some visual codes aligned ‘health’ with curative medicine, not prevention or positive maintenance. There was also a contradiction between an individualised appeal to the viewer that invoked the ‘choice’ agenda and a more traditional social medicine justification, which focused on inequalities and impersonal structures. Again these contradictions reflected their political moment, in which the value of health in pluralist consumer societies was deeply contested.

## Conclusion

Looking to the future, technology now extends the promise that digital-born sources such as those discussed here will soon be amenable to big data analysis. New data-mining software has the power to detect patterns in extensive bodies of text, which selective sampling by an individual reader could never hope to match. Visualization of the results may take basic forms, like n-gram graphs or word clouds, or more complex text-mapping, which can capture over-arching concepts and link these by pathways to subsidiary themes according to context.^61^ Grounded in reproducible quantification, the underlying results are of a different order of validity to subjective content analysis produced by the lone researcher, such as that exemplified here. Work currently underway on sources such as the Google books corpus, the digitised *BMJ* (*British Medical Journal*) and the ‘London's Pulse’ MOH Reports is showing how electronic tools can reveal hitherto unobtainable insights into the construction of medical knowledge.^62^ Political history is similarly demonstrating the capacity of text analytics to overturn received wisdom, and to provide sounder empirical argumentation in its place.^63^

In this respect, the interface of the UK Web Archive at its present stage of development points the way to what will shortly be achievable. With the full archive open, it is possible to envisage the problem of duplicates being addressed (to allow removal of all identical sites within a specified time period) and the sentiment analysis function extended (to allow readers themselves to introduce appropriate terms). Researchers will then be able speedily to create their selection of websites, assess basic features in aggregate, then (deposit regulations permitting) export the corpus for electronic text-mining using their preferred software.^64^

However, the discussion here has highlighted one vital aspect of websites that mechanised content analysis seems less suited to explicate. For they are almost always more than black type on white page, appearing instead as visual artefacts of increasing vibrancy. At minimum there are fundamentals of graphic design like colour, fonts, layout, logos and banners to consider. But even the small selection reviewed here suggests that written text is often likely to appear alongside images that augment its meaning and effect. It is gratifying to realise that the interpretive role of the researcher therefore remains irreducible, but this also carries responsibility. Historians will need to approach the website rather as they do the poster, the painting, the photograph or the film.^65^

The nature of these documents therefore means that a project concerned with health policy may end up in part an exercise in cultural studies. And indeed the sources considered here deal only with representations of the social world, revealing nothing of the outcomes of the policies they describe. Instead a sense is gained of how political languages descend through layers of government, corralling energies and legitimising action, and of how the public is constituted by health officers, with an appeal to the autonomous subject often running alongside other codes that carry strong normative assumptions about the individual and society.

As to actual effectiveness of the policies represented, there is a small specialist literature that evaluates such initiatives.^66^ This shows that health inequalities gained a stronger profile, as conditions of effective partnership working became clearer.^67^ However, evidence of measurable population health benefits resulting from ‘health improvement plans’ or ‘local strategic partnerships’ remains equivocal. To the extent that such evaluations are robust (and their authors note many confounders and challenges) studies showed that by 2009 there was ‘not yet any clear evidence of the effects of public health partnerships on health outcomes’.^68^ More generally, the 2010 Marmot Review noted that policy in the New Labour years had not narrowed health inequalities, and that some indicators—obesity prevalence, for example—had worsened.^69^ Thus, as we begin to work with websites such as these as documentary sources, it is important not to lose sight of the more important question of their relationship to lived experience.
